# Supplementary material for: GH3 Gene Family Identification in Chinese White Pear (Pyrus bretschneideri) and the Functional Analysis of PbrGH3.5 in Fe Deficiency Responses in Tomato
Source: Int J Mol Sci. 2024 Dec 3;25(23):12980. doi: 10.3390/ijms252312980 (PMC11641127; doi:10.3390/ijms252312980)
Supplement: Supplementary file 1 [file ijms-25-12980-s001.zip › Supplementary figures.pdf]

Supplementary information

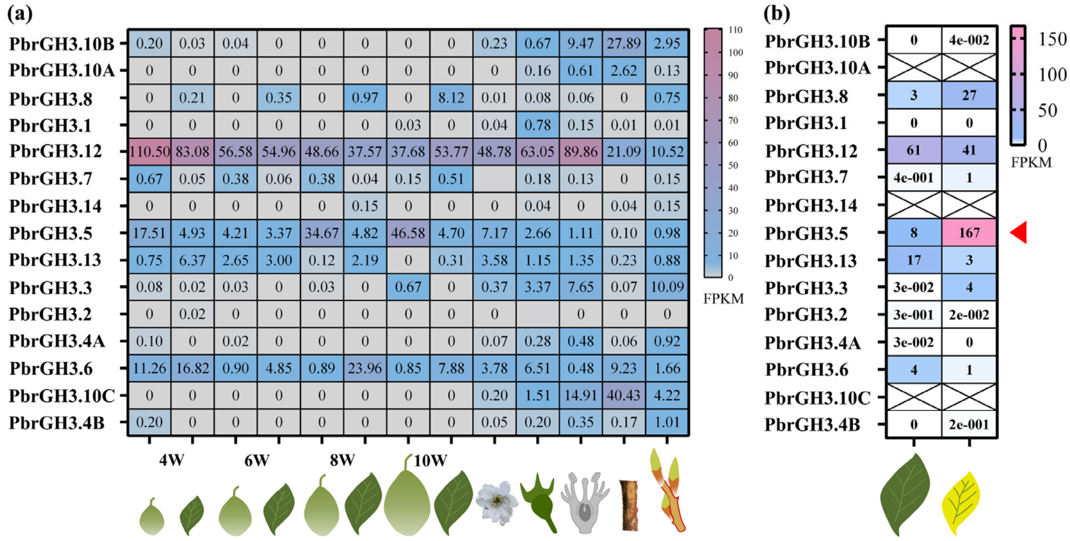

Figure S1. Expression heatmap of the *GH3* gene family in pear. **(a)** Tissue-specific expression of pear *GH3* (*PbrGH3*) genes based on the RNA-seq data of 11 samples from seven pear tissues that was retrieved on the PearMODB website (https://pearomics.njau.edu.cn/). The numbers were shown as the fragments per kilobase of transcript per million mapped reads (FPKM) values, and presented in colors. The tissues are fruits and leaves at 4 weeks, 6 weeks, 8 weeks, and 10 weeks after flower blooming, petals, calyxes, ovaries, stems, and shoots (from left to right) of 'Dangshansuli' trees. **(b)** The expression levels of *PbrGH3* genes in the normal (left, NL) and iron-deficient chlorotic leaves (right, CL) of 'Dangshansuli' trees. The data shown were based on previously established RNA-seq results and were displayed in colors. '×' in the boxes indicates not detected. The white-to-pink gradient denotes a gradual increase in expression level.

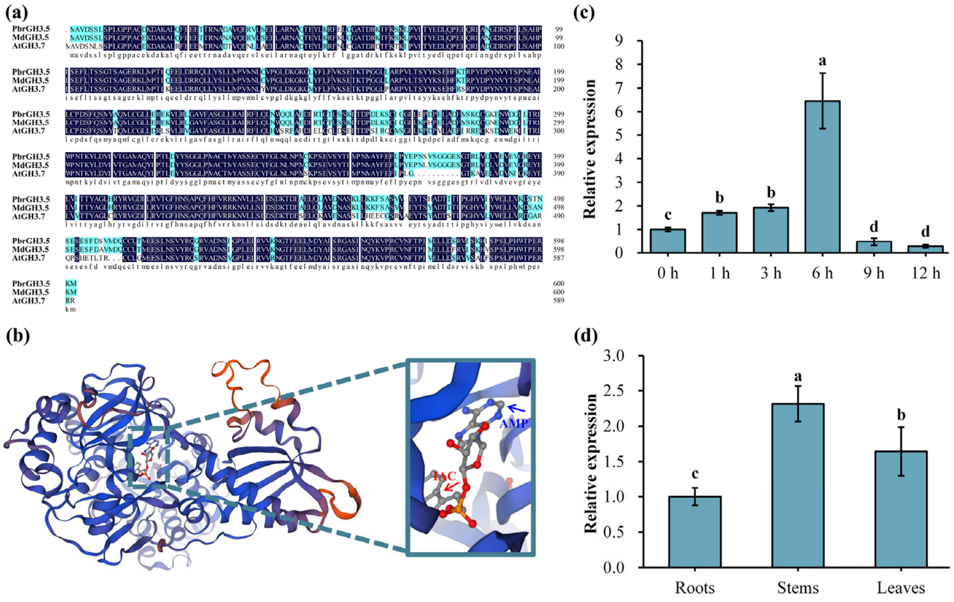

Figure S2. A brief analysis of *PbrGH3*.5 in pear. **(a)** Alignment blast of pear PbrGH3.5 protein and its homologs in arabidopsis (*Arabidopsis thaliana*, AtGH3.7) and apple (*Malus domestica*, MdGH3.5) plants. **(b)** Three-dimensional structure of PbrGH3.5 protein. Adenosine monophosphate (AMP) and 1H-indol-3-Yacetic acid (IAC) ligands were indicated by blue and red arrows, respectively. **(c)** Expression level of *PbrGH3.5* over time in leaves of pear plants stressed by Fe deficiency. The expression at 0 h was used as the control and was set to '1'. **(d)** Expression level of *PbrGH3.5* in roots, stems, and leaves of one-month-old 'Duli' (*Pyrus betulifolia*) pear seedlings. The data were shown as the mean ± standard deviation (SD) of three independent replicates (n = 3), and the statistical differences were indicated by different lowercases (a–d) at $p < 0.05$ (one-way ANOVA with Duncan's multiple range test).

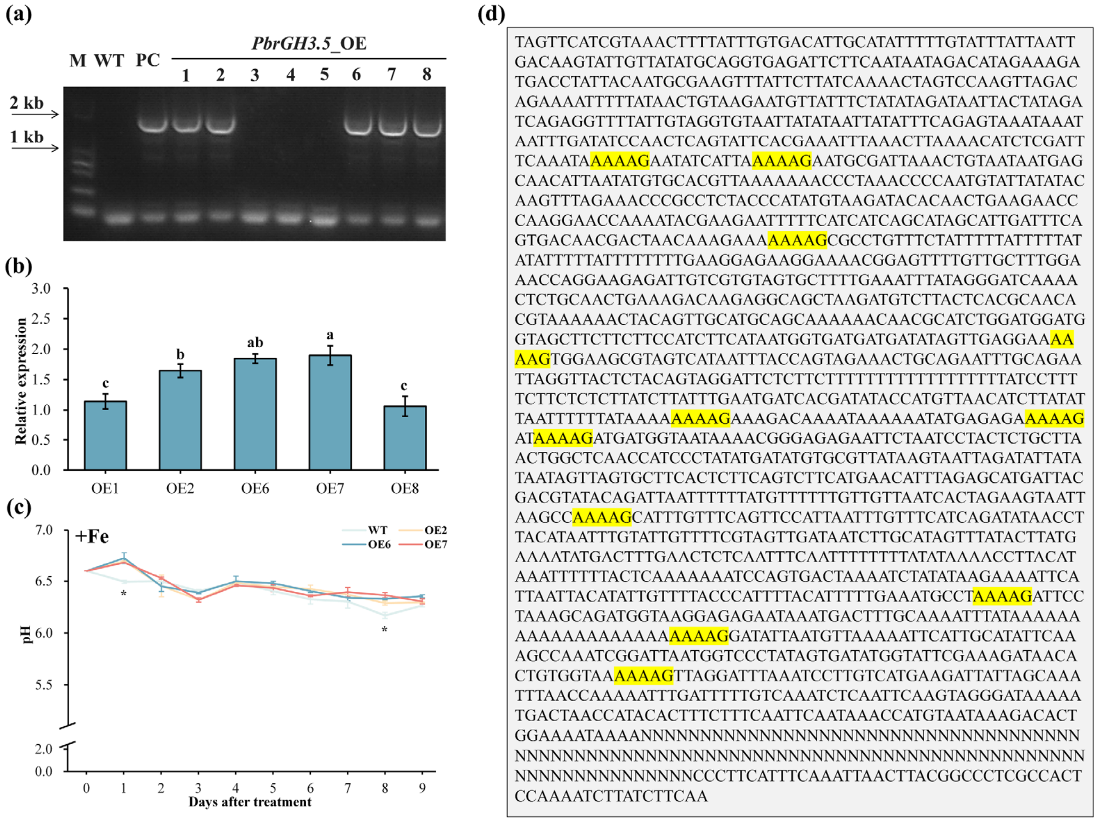

Figure S3. Molecular identification of *PbrGH3.5*-overexpressed transgenic tomato plants. **(a)** Detection of *PbrGH3.5*-overexpressing tomato plants (OE) at the DNA level. M, DNA Marker 2000; WT, negative control using the DNA of wild-type (WT) tomato plants as a template; PC, positive control using the corresponding DNAs extracted from OE plants as templates. **(b)** RT-qPCR analysis to examine the expression level of PbrGH3.5 in OE plants. The OE plant with the lowest expression of *PbrGH3.5* served as the reference, and the expression of it was set to 1.. **(c)** The rhizosphere pH over time under Fe-sufficient (+Fe) conditions. **(d)** The 2000 bp upstream region of the coding sequence of the *PbrGH3.5* gene. The potential binding sites (AAAAG) for the transcription factor Dof were labeled with yellow backgrounds. The data were shown as the mean ± standard deviation (SD) of three biological replicates (n = 3), and the statistical differences were indicated by different lowercases (a–c) or asterisks at $p < 0.05$ (one-way ANOVA with Duncan's multiple range test for charts a and b, and Student's *t*-test for chart c)

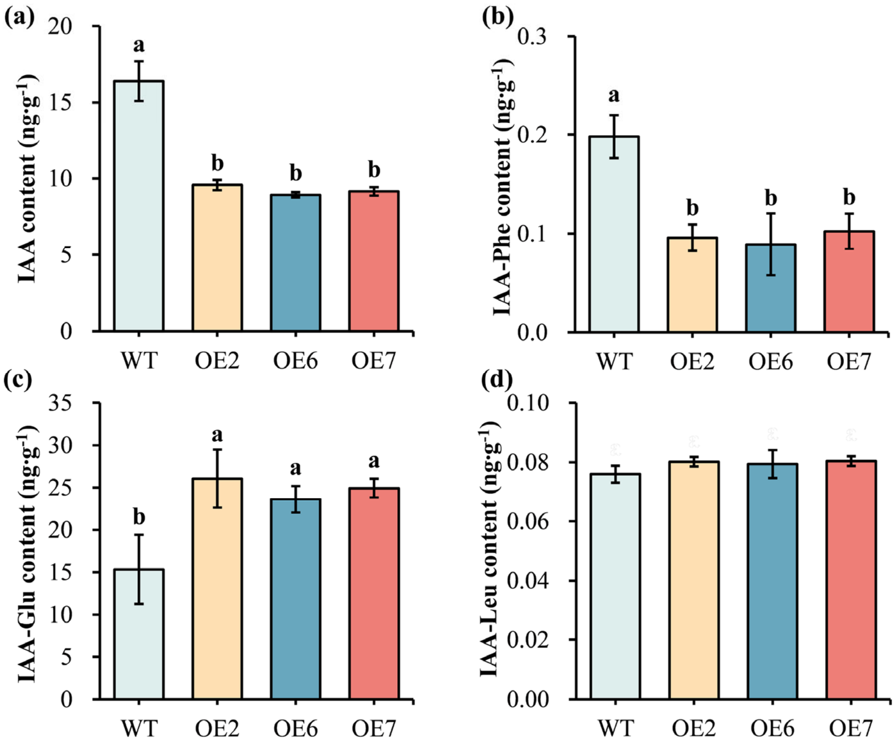

Figure S4. Ectopically overexpressing *PbrGH3.5* in tomato plants facilitates the conjugation of IAA in their leaves under Fe-deficient conditions. (a–d) The content of free IAA, IAA-phenylalanine (Phe), IAA-glutamate (Glu), and IAA-leucine (Leu) in leaves of wild-type (WT) and *PbrGH3.5*-overexpressing (OE) transgenic tomato plants after Fe deficiency treatments. The data were shown as the mean ± standard deviation (SD) of three replicates (n = 3), and the statistical differences were indicated by different lowercases (a, and b) at $p < 0.05$ (one-way ANOVA with Student's *t*-test).
